# 222. Clinical and Microbiological Characteristics of Common Bacterial Bloodstream Infections in the US Military Health System

**DOI:** 10.1093/ofid/ofab466.424

**Published:** 2021-12-04

**Authors:** Alexander C Vostal, Melissa Grance, Uzo Chukwuma, Carlos Morales, Charlotte Lanteri, Beth Poitras, Edward Parmelee, John H Powers, Katrin Mende

**Affiliations:** 1 University of Maryland Medical Center/NIAID, Silver spring, Maryland; 2 Infectious Disease Clinical Research Program, Uniformed Services University of the Health Sciences, Rockville, Maryland; 3 Navy and Marine Corps Public Health Center, Portsmouth, Virginia; 4 Uniformed Services University of the Health Sciences, Bethesda, Maryland; 5 Defense Health Agency, Falls Church, Virginia; 6 Support to National Institute of Allergy and Infectious Disease, Bethesda, MD; 7 Infectious Disease Clinical Research Program, Bethesda, MD, The Henry M. Jackson Foundation, Bethesda, MD, and Brooke Army Medical Center, Fort Sam Houston, TX, San Antonio, TX

## Abstract

**Background:**

Bloodstream infections (BSI) are associated with inpatient morbidity in the United States. We sought to characterize the epidemiology of common bacterial BSIs in individuals receiving care within the US Military Health System (MHS), which actively prospectively captures clinical and microbiological data from both retired and active-duty US Uniformed Service members and their beneficiaries.

**Methods:**

We performed a retrospective cohort study analyzing MHS patients with blood cultures positive for all bacterial pathogens, between January 2010 and December 2019. Microbiological data captured by the Navy and Marine Corpse Public Health Center, excluding cultures isolating contaminants, were retrospectively collated with clinical and demographic data from the MHS Data Repository.

**Results:**

The most frequent nine bacterial pathogens, as well as *Acinetobacter* spp. represented 17,206 episodes of BSI from 14,531 individuals. The cohort was predominantly male (59.4%) and ≥65 years old (48.7%). Most individuals were retired (N=5,249) or active duty (N=1,418) service members and their dependents (N=5,236). Median Updated Charlson Comorbidity Index Score was 2. Chronic pulmonary disease was the most frequent comorbid condition. Hospital admission was associated with 13,733 (79.8%) BSI episodes, including 5,870 admissions to the ICU. Overall, inpatient mortality was 8.3%. *E. coli* (29.7%, N= 5,114) was isolated with the highest frequency, followed by *S. aureus* (22.4%, N=3,853). Further, 9.5% of *E. coli* and 36.9% of *S. aureus* isolates were resistant to ceftriaxone and oxacillin, respectively. Beta-hemolytic streptococci represented the highest percentage (6.3%) of recurrent BSI episodes occurring at least 14 days post-initial BSI. Males or Native American race were most commonly infected with *S. aureus*. *E. coli* BSI was most common in all other demographic categories.

Frequency of Bacterial Blood Stream Infections in the US Military Health System

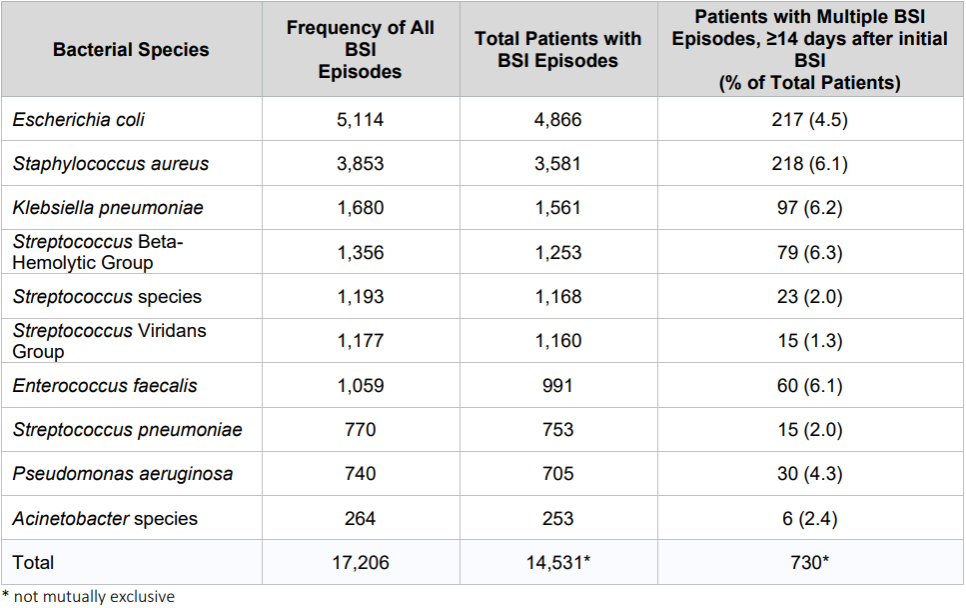

The most frequent nine bacterial pathogens, as well as Acinetobacter spp. in the US Military Health System.

**Conclusion:**

We assessed the epidemiologic features of all individuals with BSI receiving care in the MHS over a 10-year period. We noted demographic differences in the occurrence of microbiological causes of BSI including *S. aureus*. Further assessments are underway into BSI-related risk factors for occurrence, antimicrobial resistance and mortality, after controlling for comorbidities and disease severity.

**Disclosures:**

**All Authors**: No reported disclosures

